# Depressive statements prime goal-directed alcohol-seeking in individuals who report drinking to cope with negative affect

**DOI:** 10.1007/s00213-017-4765-8

**Published:** 2017-10-29

**Authors:** Lee Hogarth, Lorna Hardy

**Affiliations:** School of Psychology, University of Exeter, Washington Singer Building, Perry Road, Exeter, EX4 4QG UK

**Keywords:** Incentive learning, Goal-directed learning, Habit, Drug-seeking, Negative mood, Depression, Allostasis, Negative reinforcement, Alcohol

## Abstract

**Background:**

Most variants of negative reinforcement theory predict that acute depressed mood can promote alcohol-seeking behaviour, but the precise mechanisms underpinning this effect remain contested. One possibility is that mood-induced alcohol-seeking is due to the formation of a stimulus-response (S-R) association, enabling depressed mood to elicit alcohol-seeking automatically. A second possibility is that depressed mood undergoes incentive learning, enabling it to enhance the expected value of alcohol and thus promote goal-directed alcohol-seeking.

**Objectives:**

These two explanations were distinguished using a human outcome-revaluation procedure.

**Methods:**

One hundred and twenty-eight alcohol drinkers completed questionnaires of alcohol use disorder, drinking to cope with negative affect and depression symptoms. Participants then learned that two responses earned alcohol and food points respectively (baseline) in two alternative forced choice trials. At test, participants rated the valence of randomly sampled negative and positive mood statements and, after each statement, chose between the alcohol- and food-seeking responses in extinction.

**Results:**

The percentage of alcohol- versus food-seeking responses was increased significantly in trials containing negative statements compared to baseline and positive statement trials, in individuals who reported drinking to cope with negative affect (*p* = .004), but there was no such interaction with indices of alcohol use disorder (*p* = .87) or depression symptoms (*p* = .58).

**Conclusions:**

Individuals who drink to cope with negative affect are more sensitive to the motivational impact of acute depressed mood statements priming goal-directed alcohol-seeking. Negative copers’ vulnerability to alcohol dependence may be better explained by excessive affective incentive learning than by S-R habit formation.

## Introduction

The core tenet of negative reinforcement theory is that alcohol dependence is caused by withdrawal, emotional, or psychiatric states (such as agitation, depression, and anxiety) powerfully motivating alcohol use in order to mitigate these states (Baker et al. 2004; Cox and Klinger 1988; Eissenberg 2004; Hall et al. 2015; Kassel et al. 2003; Khantzian 1997; Koob and Volkow 2010; Marlatt 1996; Mathew et al. 2017; Sinha 2001; Solomon and Corbit 1973; Wikler 1984). However, the exact mechanisms by which adverse states trigger alcohol-seeking remain unclear. Several negative reinforcement accounts claim that negative affect triggers alcohol-seeking automatically, i.e. without forethought for the consequences (Baker et al. 2004; Everitt and Robbins 2016; Koob and Volkow 2010; Schwabe et al. 2011). This claimed automatic status of alcohol-seeking arguably explains why drinking persists despite significant harms or intentions to quit. These theoretical papers articulate two variants of the automatic account. According to one variant, alcohol’s ability to mitigate adverse states means that alcohol is experienced as having a greater reward value in those states. The greater reward value of alcohol reinforces a strong direct association (connection) between the adverse state stimuli (S) and the alcohol-seeking motor response (R). These stimulus-response (S-R) links enable the adverse states to elicit the alcohol-seeking response automatically, unconsciously, habitually, or compulsively, i.e. without forethought for the wider harmful consequences of alcohol use or current intentions to quit. The second variant of the automatic account differs in that it presumes that adverse states (e.g. anxiety) acutely reduce cognitive capacity, favouring automatic control over alcohol-seeking by S-R links which have previously formed between *external* alcohol-related stimuli and the alcohol-seeking response. By promoting automatic control over alcohol-seeking by external alcohol cues, adverse states reduce the influence of expected harms and intentions to quit on behaviour, and so promote dependence and relapse.

Other negative reinforcement theories, by contrast, claim that adverse affective states motivate alcohol-seeking by retrieving explicit coping motives—beliefs that alcohol can help mitigate adverse states (Cox and Klinger 1988; Kassel et al. 2003; Khantzian 1997; Marlatt 1996; Mathew et al. 2017; Sinha 2001). Such motivational negative reinforcement models may be specified in more mechanistic detail by being integrated with incentive learning theory (Dickinson and Balleine 2010; Hogarth et al. 2015; Hutcheson et al. 2001). According to this combined account, individuals who report drinking to cope with negative affective states are reporting their direct experience alcohol having a greater reward value because of its ability to acutely mitigate those states. This incentive learning experience enables negative affective states to raise the expected value of alcohol (in the same way that hunger raises the expected value of food, because food is more rewarding when hungry). The greater expected reward value of alcohol in the negative affective state is combined with instrumental knowledge of the responses that produce alcohol in the current environmental context (Hardy et al. 2017), thus promoting goal-directed (intentional) instrumental choice to obtain alcohol. In short, individuals who report drinking to cope with negative affect are vulnerable to alcohol dependence and relapse (Beseler et al. 2008; Crum et al. 2013a, b; Lazareck et al. 2012; Menary et al. 2011; Merrill et al. 2014; Robinson et al. 2011; Windle and Windle 2015), because negative affective states act as powerful motivators of goal-directed alcohol-seeking which overrule expected harms and intentions to quit (just as intense hunger might overrule weight loss intentions).

A key source of evidence supporting this particular incentive learning model is the finding that individuals who report drinking to cope with adverse affective states are more sensitive to the motivational impact of experimentally induced negative mood or stress on alcohol-seeking behaviour, as indexed by craving, consumption, preferential choice, or cognitive bias (Austin and Smith 2008; Birch et al. 2004; Brady et al. 2006; Cooney et al. 1997; Field and Powell 2007; Field and Quigley 2009; Grant et al. 2007; Rousseau et al. 2011; Woud et al. 2015; Zack et al. 2003); but for null results, see (Field and Powell 2007; Thomas et al. 2014). The strong interpretation of these findings is that coping motives play a causal role in enabling mood induction to promote alcohol-seeking, rather than automatic S-R processes. However, because coping motives are only correlated with mood-induced alcohol-seeking, they could be merely epiphenomenal, while an S-R process is actually responsible for the effect. Existing studies cannot discriminate these two positions.

The outcome-revaluation procedure has provided a more decisive method for determining whether drug-seeking behaviour is controlled by incentive learning or S-R mechanisms, in both animals (Corbit et al. 2012; Dickinson et al. 2002; Hutcheson et al. 2001; Miles et al. 2003) and humans (Hogarth 2012; Hogarth and Chase 2011; Hogarth et al. 2013). The rationale of this method can be illustrated with one key study. Hutcheson et al. (2001) found that heroin withdrawal could augment a novel heroin-seeking response in an extinction test, but only in rats that had previously experienced heroin in the withdrawal state. This effect can be explained by incentive learning but not by S-R theory. Arguably, rats learn that heroin has greater reward value in the withdrawal state (incentive learning), enabling this state to raise the expected value of heroin, which integrates with instrumental knowledge of the novel heroin-seeking response, enabling goal-directed selection of that response. By contrast, S-R mechanisms cannot explain this effect for two reasons. First, the heroin-seeking response was never reinforced in the withdrawal state, so an S-R association could not form between withdrawal and the response. The other S-R variant is also not viable, because if withdrawal impaired cognition promoting control over heroin-seeking by S-R links between external cues and the response, then withdrawal should have promoted heroin-seeking in rats that had not previously experienced heroin in that state (had no incentive learning experience). However, this effect was not found. Thus, the outcome-revaluation procedure provides a compelling test of whether the impact of negative affective states on drug-seeking behaviour is driven by incentive learning rather than S-R mechanisms.

The current study utilised a human outcome-revaluation procedure to test whether acute depressed mood statements would prime goal-directed alcohol-seeking to a greater extent in individuals who report drinking to cope with negative affect. One hundred and twenty-eight alcohol drinkers first completed questionnaires of alcohol use disorder, drinking to cope with negative affect and depression symptoms. Participants then learned at baseline that two responses earned alcohol and food points respectively in a set of two alternative forced choice trials. At test, participants rated the valence of randomly sampled negative affective statements (e.g. ‘I don’t think things are ever going to get better’) and positive statements (e.g. ‘I feel enthusiastic and confident now’), and following each statement, chose between the alcohol- and food-seeking responses in extinction (i.e. no alcohol or food points were earned). It was expected that negative mood statements would increase the percentage of alcohol- versus food-seeking choices compared to positive statements and baseline to a greater extent in individuals who report drinking to cope with negative affect. This finding would support a merger of motivational negative reinforcement theory (Cox and Klinger 1988; Kassel et al. 2003; Khantzian 1997; Marlatt 1996; Mathew et al. 2017; Sinha 2001) and incentive learning theory (Dickinson et al. 2002; Hogarth 2012; Hogarth and Chase 2011; Hutcheson et al. 2001). That is, the finding would suggest that explicit beliefs concerning the greater reward value of alcohol in the negative affective state are the causal mechanism driving the intentional choice to drink, rather than an automatic S-R mechanism. This theoretical distinction has important implications for alcohol treatment strategy, suggesting that for drinkers who report negative coping motives, the most effective treatment should be forms of cognitive behaviour therapy (CBT) that directly target negative coping motives (Anker et al. 2016; Bradizza et al. 2017; Kushner et al. 2013; Stasiewicz et al. 2013), whereas mood management (Monti et al. 1990; Monti and Rohsenow 1999; Pettinati et al. 2013), and attempts to counter-train implicit learning processes (Gladwin et al. 2015) are likely to be comparatively less effective in this group.

## Method

### Participants

The study recruited 128 drinkers (50% male) who reported drinking alcohol at least monthly. The study was approved by the University of Exeter Psychology Ethics Committee.

### Questionnaires

Questionnaires were the alcohol use disorders inventory test (AUDIT: Babor et al. 2001) and the Reasons for Drinking Questionnaire (RFDQ: Zywiak et al. 1996) from which the negative coping subscale was examined. This subscale includes seven items which ask participants to assess how important different reasons for drinking are for them, including sadness, anger, frustration, anxiety, tension, illness, and relationship difficulties, measured on a 0–10 scale ranging from ‘not at all important’ to ‘very important’. Depression symptoms were recorded using Beck’s Depression Inventory II (Beck et al. 1996).

### Mood induction effect on alcohol choice

**Fig. 1:**
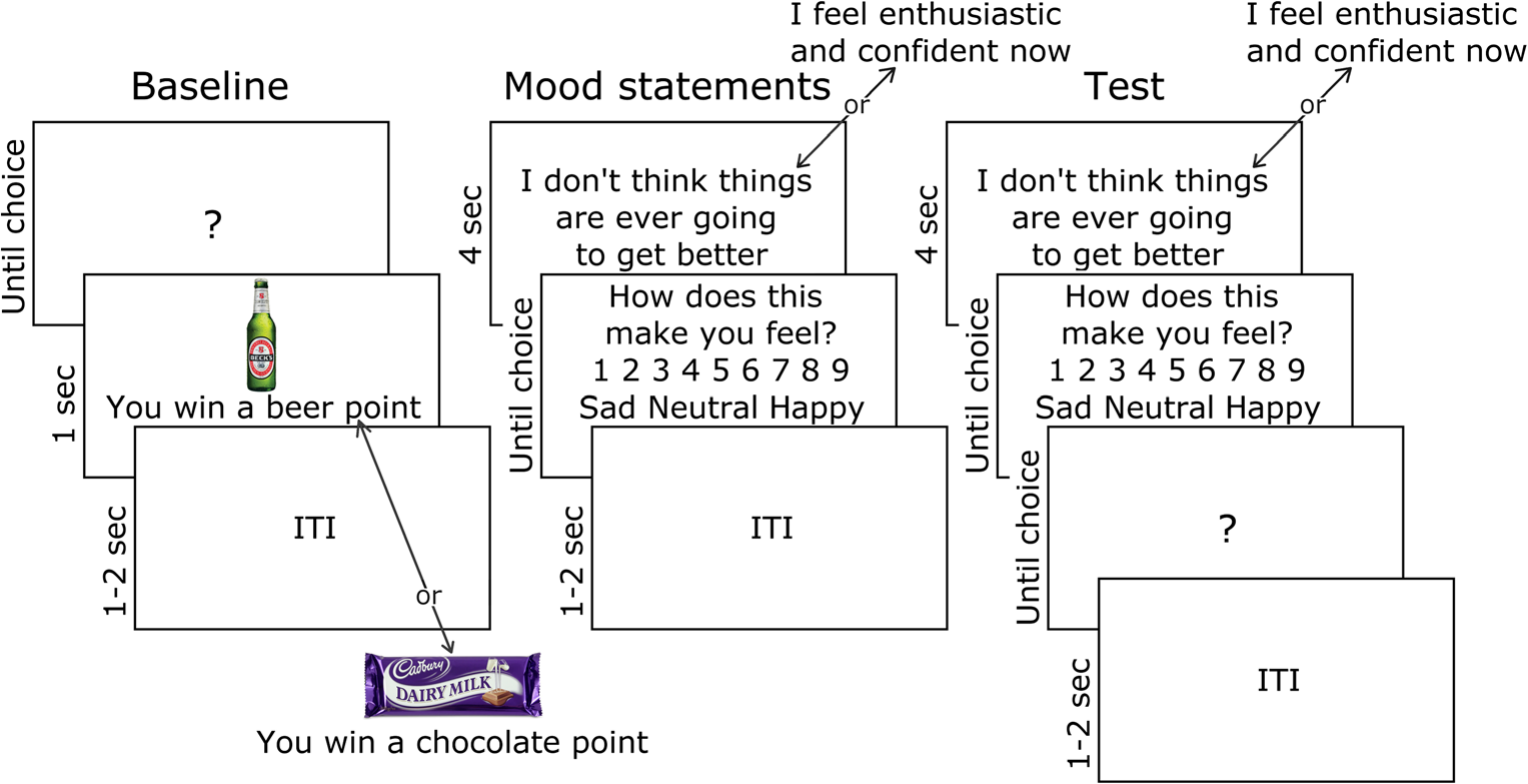
Outcome-revaluation procedure used to test the impact of negative and positive mood statements on goal-directed alcohol-seeking. At baseline, participants learned that left and right keyboard responses earned beer and chocolate points, respectively. Participants then rated how sad or happy randomly sampled negative and positive mood statements made them feel (see Table 1 for a list of statements). At test, participants continued to rate the valence of negative and positive statements, but after each statement, made a free choice between the beer- or chocolate-seeking response trained at baseline, but without feedback of whether beer or chocolate points were earned (i.e. in extinction). Negative mood statements were expected to increase the percentage of beer- over chocolate-seeking responses, compared to positive statements and baseline, in individuals who reported drinking to cope with negative affect. This would demonstrate greater sensitivity to the motivational effect of negative mood statements on goal-directed alcohol-seeking

Baseline alcohol versus food choice (see Fig. 1): participants were presented with two 275-ml bottles of Beck’s beer and two 45-g Cadbury’s Dairy Milk chocolate bars on the desk and instructed: ‘In this task, you can earn points for beer and chocolate to take with you at the end. In each trial, choose the UP or DOWN arrow key to earn these rewards. Your points will be drawn from a lottery at the end of the experiment. You may win the 2 beers, the 2 chocolate bars, all 4 or none at all. The more points you earn for each reward, the better your chances of winning more of that reward’. This was a deception—all participants received a small Freddo chocolate bar at the end of the experiment. Trials began with a question mark, whereupon an up or down key press produced the alcohol or chocolate outcome, comprising an image of the reinforcer plus corresponding text ‘You win a [beer/chocolate] point’ for 1 s. The response-outcome contingencies were counterbalanced between subjects. After 32 baseline trials, contingency knowledge was tested with two questions in random order: ‘Which arrow key earned [beer/chocolate] the UP or DOWN key?’

Mood statements: participants were instructed to carefully consider negative and positive mood statements listed in Table 1 (Hogarth et al. 2015; Velten 1968; Westermann et al. 1996). In each trial, either a negative or positive statement was presented for 4 s, before participants rated how sad to happy this made them feel on a 9-point scale. Across eight trials, the presented statement was randomly selected from the set of 32, comprising 16 negative and 16 positive statements (see Table 1).

**Table 1:** Negative and positive mood statements used in the study. At the beginning of each test trial, one statement was presented (randomly sampled from the entire set of 32), and rated for how sad-happy it makes the participant feel, before a choice was made between the alcohol- or food-seeking response in extinction

Negative mood statements	Positive mood statements
• I feel a little down today	• I feel cheerful and lively
• My work is harder than I expected	• On the whole, I have very little difficulty in thinking clearly
• Sometimes I feel so guilty that I can’t sleep	• I’m pleased that most people are so friendly to me
• I wish I could be myself, but nobody likes me when I am	• I can make friends extremely easily
• Today is one of those days when everything I do is wrong	• I feel enthusiastic and confident now
• I doubt that I’ll ever make a contribution in the world	• There should be a lot of good times coming along
• I feel like my life is in a rut that I’m never going to get out	• I’m able to do things accurately and efficiently
• My mistakes haunt me, I’ve made too many	• I know that I can achieve the goals I set
• Life is such a heavy burden	• I have a sense of power and vigour
• I’m tired of trying	• I’m feeling amazingly good today
• Even when I give my best effort, it just doesn’t seem to be good enough	• I feel highly perceptive and refreshed
• I don’t think things are ever going to get better	• I can concentrate hard on anything I do
• I feel worthless	• My thinking is clear and rapid
• What’s the point of trying	• Life is so much fun; it seems to offer so many sources of fulfilment
• I feel cheated by life	• Life is firmly in my control
• Every time I turn around, something else has gone wrong	• I’m really feeling sharp now

Test: participants were instructed: ‘In this part of the task, please continue to consider the mood statements. Also, the UP and DOWN arrow keys will win beer and chocolate points in the same way as earlier in the task. You will be told how many points you have earned at the end. Press the space bar to begin’. In each test, a mood statement was presented for 4 s, before participants rated how sad-happy it made them feel. Upon presentation of the question mark, the alcohol- or food-seeking response was made. No outcomes were presented at the test stage, so any effect of mood statements on choice must be mediated by goal-directed knowledge of the response-outcome contingencies acquired in training. Across 64 test trials, there were two cycles of 32, each containing 16 negative and 16 positive statements selected in random order. Retention of contingency knowledge over the test phase was tested as before.

### Analytical plan

Percent alcohol- versus food-seeking choice was calculated from baseline trials and test trials with negative and positive statements (> 50% = preference for alcohol, < 50% = preference for food). An ANOVA first tested the difference between these three conditions. Separate general linear models (GLMs) were conducted with percent alcohol choice as the dependent variable, condition (3) as the within-subjects variable and a single, continuous between-subjects variable in each model: AUDIT, negative coping motives (RFDQ), and depression symptoms (BDI). A significant interaction indicated that the difference in alcohol choice between conditions varied with the continuous variable. A main effect of the continuous variable indicated that there was a simple correlation between overall alcohol choice and the continuous variable. Interactions were followed up by GLMs contrasting the three conditions.

## Results

### Participants

Of the 128 participants recruited, five failed to accurately report the response-outcome contingencies after baseline or test and so were excluded, as is standard in this paradigm (e.g. Hogarth et al. 2015). The mean characteristics of the 123 participants who were analysed were as follows: age = 20.9 (SD = 1.7, range = 18–32), AUDIT = 10.2 (5.1, 1–25), RFDQ negative coping score = 1.7 (1.5, 0–5.9), and BDI = 5.7 (5.4, 0–26). There were 61 males and 62 females.

### Experimental task

Negative and positive statements were rated as having significantly different valence. The negative mood statements were rated as making participants feel sad (*M* = 2.71, *SEM* = 0.09), whereas the positive statements were rated as making participants feel happy (*M* = 7.36, *SEM* = 0.09), *F*(1122) = 1036.42, *p* < .001, η_p_^2^ = .895. There was no overall difference in alcohol choice measured between the baseline, test-negative, and test-positive conditions, *F*(1122) = 1.14, *p* = .32, η_p_^2^ = .009, as shown in Fig. 1a.

**Fig. 2:**
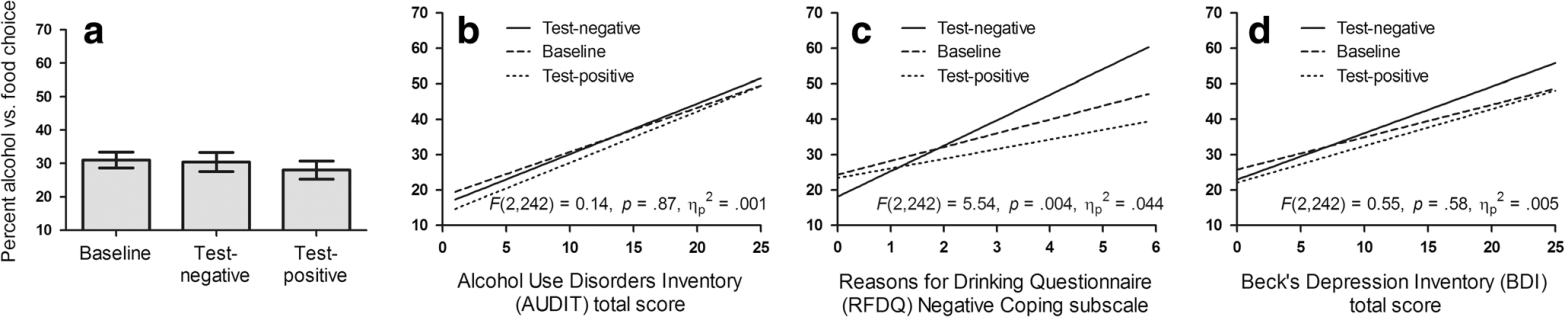
**a** Average percent choice of the alcohol- over food-seeking response in baseline, test-negative, and test-positive trials. **b**–**d** Regression slopes relating the percent choice of alcohol- over food-seeking responses in baseline, test-negative, and test-positive trials with three continuous between-subjects variables: **b** alcohol use disorder AUDIT scores, **c** the Reasons for Drinking Questionnaire negative coping subscale, and **d** Beck’s Depression Inventory. The statistical insets report the interaction between the within-subjects variable condition (3) and the continuous between-subjects variable AUDIT, RFDQ, or BDI. The significant interaction involving RFDQ negative coping (**c**) indicates that individuals who reported drinking to cope with negative affect were more sensitive to the motivational impact of negative mood statements on goal-directed alcohol-seeking

Table 2 shows the correlation matrix between the questionnaire scales, baseline alcohol versus food choice, and the mood-induced increase in alcohol choice (the difference between test-negative and test-positive conditions). The GLM with AUDIT shown in Fig. 2b showed a significant main effect of AUDIT, *F*(1121) = 9.52, *p* = .003, η_p_^2^ = .073 but no interaction between AUDIT and condition, *F*(2242) = 0.14, *p* = .87, η_p_^2^ = .001. By contrast, the GLM with RFDQ negative coping shown in Fig. 2c revealed a significant main effect of RFDQ negative coping, *F*(1121) = 8.93, *p* = .003, η_p_^2^ = .069, and significant interaction between RFDQ negative coping and condition, *F*(2242) = 5.54, *p* = .004, η_p_^2^ = .044. Specific contrasts indicated that this interaction between RFDQ negative coping and condition was significant when the GLM included baseline and test-negative conditions, *F*(1121) = 7.33, *p* = .008, η_p_^2^ = .057, and when it included test-negative and test-positive conditions, *F*(1121) = 7.44, *p* < .007, η_p_^2^ = .058, but not when it included baseline and test-positive conditions, *F*(1121) = 0.82, *p* = .37, η_p_^2^ = .007. Finally, the GLM with BDI shown in Fig. 2d revealed a main effect of BDI, *F*(1121) = 6.50, *p* = .01, η_p_^2^ = .051, but no interaction with between BDI and condition, *F*(2242) = 0.55, *p* = .58, η_p_^2^ = .005. In summary, these results indicate AUDIT, RFDQ negative coping, and BDI all have a simple correlation with baseline and overall alcohol choice, but only RFDQ negative coping is associated with greater sensitivity to the motivational impact of negative mood statements on alcohol-seeking at test.

**Table 2:** Correlation matrix between questionnaire and alcohol-seeking measures

	Experiment 1			
	BDI	RFDQ negative coping	Baseline alcohol choice	Mood induced alcohol choice
AUDIT	*r* = .25 *p* = .005	*r* = .43 *p* < .001	*r* = .24 *p* = .007	*r* = − .00 *p* = .967
BDI		*r* = .43 *p* < .001	*r* = .19 *p* = .038	*r* = .05 *p* = .553
RFDQ negative coping			*r* = .22 *p* = .015	*r* = .24 *p* = .007
Baseline alcohol choice				*r* = .07 *p* = .461

Baseline alcohol choice was the percent choice of alcohol over food at baseline. Mood-induced alcohol choice was the difference in percent alcohol choice between the test-negative and test-positive conditions

*AUDIT* alcohol use disorders inventory, *RFDQ* reasons for drinking questionnaire, *BDI* Beck’s Depression Inventory

Secondary analyses were undertaken to explore the role of other variables. A GLM incorporating the second RFDQ subscale, social pressure, and condition (baseline, test-negative, test-positive) revealed no main effect of subscale, *F*(1121) = 3.62, *p* = .06, η_p_^2^ = .029, or interaction, *F*(2242) = 2.68, *p* = .07, η_p_^2^ = .022. Similarly, a GLM incorporating the remaining RFDQ subscale, cued craving, and condition (3) revealed a significant main effect of subscale, *F*(1121) = 7.93, *p* = .006, η_p_^2^ = .062, but no interaction, *F*(2242) = .42, *p* = .66, η_p_^2^ = .003. Importantly, the interaction between RFDQ negative coping and condition (3) remained significant when the other two RFDQ subscales were both included (controlled) in the GLM, *F*(2238) = 6.83, *p* = .001, η_p_^2^ = .054. Similarly, the interaction between RFDQ negative coping and condition (3) remained significant when BDI and AUDIT were both included (controlled) in the model, *F*(2238) =6.39, *p* = .002, η_p_^2^ = .051. Turning to the gender variable, an ANOVA incorporating gender (2) and condition (3) indicated that males chose more alcohol overall (M = 34.8, SEM = 3.3) than females (M = 24.8, SEM = 3.3), *F*(1121) = 4.58, *p* = .03, η_p_^2^ = .037, but there was no interaction between gender and condition, *F*(2242) = .75, *p* = .47, η_p_^2^ = .006. A GLM incorporating age and condition (3) revealed no effect of age, *F*(1121) = 2.33, *p* = .13, η_p_^2^ = .019, or interaction, *F*(2242) = .19, *p* = .83, η_p_^2^ = .002. A GLM incorporating the difference in valence rating between negative and positive statements and condition (3) revealed no effect of valence rating, *F*(1121) = .37, *p* = .54, η_p_^2^ = .003, or interaction, *F*(2242) = 1.13, *p* = .32, η_p_^2^ = .009. These results indicate that the relationship between RFDQ negative coping and greater sensitivity to mood-induced alcohol-seeking cannot be explained by other variables measured in the study.

## Discussion

The main finding of the current study was that individuals who reported drinking to cope with negative affect were more sensitive to the motivational impact of negative mood statements promoting goal-directed alcohol- versus food-seeking in an outcome-revaluation procedure. This finding advances previous studies which have also found that coping motives predict sensitivity to mood or stress-induced alcohol-seeking, as indexed by craving, consumption, preferential choice, or cognitive bias (Austin and Smith 2008; Birch et al. 2004; Brady et al. 2006; Cooney et al. 1997; Field and Powell 2007; Field and Quigley 2009; Grant et al. 2007; Rousseau et al. 2011; Woud et al. 2015; Zack et al. 2003) and disconfirms two null results (Field and Powell 2007; Thomas et al. 2014). The novel contribution of the current study was to demonstrate that mood-induced alcohol-seeking can be driven by incentive learning rather than S-R habit processes. Previous studies could not distinguish these accounts. According to the incentive learning account, individuals who reported negative coping motives have learned that alcohol is more rewarding in negative affect states, enabling negative statements to raise the expected value of alcohol, which is integrated with instrumental knowledge of which response produces alcohol, promoting goal-directed choice of that response. This finding supports a merger of motivational negative reinforcement theories (Cox and Klinger 1988; Kassel et al. 2003; Khantzian 1997; Marlatt 1996; Mathew et al. 2017; Sinha 2001) and incentive learning theory (Dickinson et al. 2002; Hogarth 2012; Hogarth and Chase 2011; Hutcheson et al. 2001), in which explicit beliefs concerning the greater reward value of alcohol in the negative affective state are the causal mechanism driving the intentional choice to drink, rather than an automatic S-R mechanism, in individuals who report negative drinking coping motives.

The putative causal role played by negative coping motives and accompanying sensitivity to mood-induced alcohol-seeking in alcohol dependence and relapse has been supported by a range of studies. In longitudinal studies, self-reported coping motives are a prospective marker for subsequent alcohol dependence (Beseler et al. 2008; Crum et al. 2013a; Crum et al. 2013b; Lazareck et al. 2012; Menary et al. 2011; Merrill et al. 2014; Robinson et al. 2011; Windle and Windle 2015). For instance, Crum et al. (2013b) found that, for individuals who reported drinking to cope with negative affect at baseline, there was a 3.1 times increase in risk of new-onset alcohol dependence and a 3.4 times increased risk of persistent alcohol dependence at follow-up. Second, in cross-sectional studies, a wide range of psychiatric symptoms are associated with more severe alcohol dependence, and this relationship is consistently mediated by self-reported drinking to cope with negative affect, suggesting coping motives are the proximal driver of alcohol dependence (Asberg and Renk 2012; Dvorak et al. 2014; Fossos et al. 2011; Gonzalez et al. 2011; Grayson and Nolen-Hoeksema 2005; Holahan et al. 2001; Kaysen et al. 2007; McDevitt-Murphy et al. 2015; Mooney et al. 2008; O’Hare and Sherrer 2011; Øverup et al. 2015; Peirce et al. 1994; Reardon et al. 2002; Schuck and Widom 2001; Schuckit et al. 2006; Simpson et al. 2014; Stewart et al. 2001; Topper et al. 2011; Ullman et al. 2005; Yeater et al. 2010; Young-Wolff et al. 2009). Third, retrospective interview studies have found that alcoholics typically attribute more than 50% of relapse episodes to negative affect, interpersonal conflict, or physical ailments, suggesting that reactivity to negative triggers drives relapse (Brown et al. 1990; Hodgins et al. 1995; Hore 1971; Marlatt 1996). Finally, greater increases in alcohol craving following experimental negative mood induction predicts vulnerability to alcohol relapse even when other relevant predictors are controlled (Brady et al. 2006; Cooney et al. 1997; Higley et al. 2011; Sinha et al. 2011) also for cocaine relapse, see (Back et al. 2010; Sinha et al. 2006). For example, Sinha et al. (2011) found that only 0.02% of high stress-induced craving responders remained abstinent from alcohol at an 80-day follow-up, whereas 35% of low stress-induced craving responders survived. These studies are consistent with the claim that explicit beliefs that alcohol has a greater reward value in a negative affect state (incentive learning) plays a causal role in driving alcohol-seeking behaviour.

By contrast, the main finding cannot be explained by S-R accounts of how depressed mood promotes alcohol-seeking (Baker et al. 2004; Everitt and Robbins 2016; Koob and Volkow 2010; Schwabe et al. 2011). Negative mood statements could not have formed a stronger S-R association with the alcohol- versus food-seeking response, because testing was conducted in extinction and, therefore, neither response was reinforced in the presence of negative mood statements. Similarly, external contextual cues were commonly present when both responses were made during baseline training, and so would have formed equivalent S-R links with the alcohol- and food-seeking responses. Consequently, negative statements could not have promoted alcohol-seeking through either a stronger S-R link to that response, or by facilitating S-R links between external cues and the alcohol-seeking response. Finally, S-R theory could only explain the correlation between coping motives and mood-induced alcohol-seeking by suggesting that coping motives are epiphenomenal rather than causal, which contradicts substantial data demonstrating the importance of coping motives in alcohol dependence noted above.

There are implications for treatment strategy in the concluding that negative mood-induced alcohol-seeking in those who drink to cope is driven by incentive learning rather than more automatic S-R mechanisms. First, if the belief that alcohol has a higher value in negative affect states plays a causal role in driving alcohol-seeking in individuals who drink to cope, then CBT which targets coping motives should be most effective in this group. Support for this claim comes from the finding that versions of CBT that target negative coping motives are more effective than treatment as usual (Bradizza et al. 2017; Chaney et al. 1978; Jones et al. 1982; Kushner et al. 2013; Monti et al. 1990; Stasiewicz et al. 2013; Watt et al. 2006), and this therapeutic effect is greater in individuals who report negative coping motives (Anker et al. 2016). Second, brief interventions which target coping motives have also produced promising therapeutic outcomes. For example, Conrod et al. (2013) selected high-risk adolescents who were high in anxiety, hopelessness, impulsivity, or sensation-seeking and trained them to identify individualised drinking triggers and adaptive coping strategies. This intervention reduced the odds of drinking during the trial by 29% compared to no treatment, suggesting that targeting coping motives in high-risk individuals may function as an effective preventative strategy. Similarly, Blevins and Stephens (2016) found that in undergraduates drinkers, a single session focusing on negative drinking coping motives and alternative coping strategies (in contrast to normative alcohol education) reduced self-reported drinking problems at a 2-month follow-up, which was mediated by reductions in drinking coping motives (see also Banes et al. 2014). Finally, trait adaptive coping skills have been shown to protect drinkers who reported drinking to cope, from stress-induced priming of alcohol consumption (Merrill and Thomas 2013), and to be associated with reduced negative coping motives and alcohol use problems (Bravo et al. 2016; Fernandez et al. 2010; Littlefield et al. 2010; Murphy and Mackillop 2012; Pearson et al. 2015; Roos et al. 2015; Tull et al. 2015). The implication of these studies is that CBT which targets negative coping motives is potentially the optimal treatment strategy for individuals who drink to cope with negative affect, consistent with the incentive learning account. By contrast, mood management (Monti et al. 1990; Monti and Rohsenow 1999; Pettinati et al. 2013) and attempts to counter-train implicit learning processes (Gladwin et al. 2015) should be comparatively less effective, because they do not tackle the beliefs that drive alcohol-seeking this group.

One important issue for the incentive learning account is whether the motivational impact of adverse states on goal-directed drug-seeking is powerful enough to override the catastrophic costs of drug use and intentions to quit—the hallmark of dependence. Two studies suggest that this is possible. First, Hutcheson et al. (2001) showed that heroin withdrawal could promote goal-directed heroin-seeking. Given that alcohol withdrawal constitutes severe and diverse symptoms, including seizures, delirium tremens, anxiety, depression, and sleep disturbance (Heilig et al. 2010), it is plausible that these states (or anticipation of them) would exert a sufficiently powerful motivating effect on goal-directed alcohol-seeking to override costs and intentions to quit. Second, we recently demonstrated using a similar outcome-revaluation procedure to that in the present study, that negative mood induction increased goal-directed tobacco-seeking even in smokers who were tobacco sated, and who would otherwise reduce their tobacco-seeking when mood induction was absent (Hogarth et al. 2015). The implication is that negative mood acted as a powerful motivational state which was capable of fully overriding satiety, and might therefore plausibly be able to override expected harms and intentions to quit.

Mood-induced alcohol-seeking did not vary with either AUDIT or BDI scores, despite these scores correlating with RFDQ negative coping (these correlations have also been reported in other studies: Armeli et al. 2010; Bolton et al. 2009; Bravo et al. 2016; Cooper et al. 1995; Gonzalez et al. 2009, 2011; Grant et al. 2009; Holahan et al. 2004; Peirce et al. 1994; Rafnsson et al. 2006; Turner et al. 1997). Furthermore, the relationship between mood-induced alcohol-seeking and RFDQ negative coping remained significant when AUDIT and BDI scores were controlled, consistent with the view that coping motives are the proximal determinant of the mood induction effect (Cooper et al. 1995; Hufford et al. 2003; Marlatt 1985; Shiffman 2005; Witkiewitz et al. 2007; Zack et al. 1999). In contrast, some studies have found that sensitivity to mood-induced alcohol-seeking increased with alcohol dependence and depression symptoms. Specifically, three studies found that mood-induced alcohol-seeking was greater in more dependent drinkers (Sinha et al. 2009; Zack et al. 2003; Zack et al. 2006), but several others have either reported nonsignificant associations (Austin and Smith 2008; Cooney et al. 1997; Field and Powell 2007; Field and Quigley 2009; Woud et al. 2015; Zack et al. 1999) or have not reported the analysis despite having the relevant data (Birch et al. 2004; Grant et al. 2007; McGrath et al. 2016; Owens et al. 2014; Potthast et al. 2015; Rousseau et al. 2011). With respect to depression symptoms, two studies have shown that the correlation between depression symptoms and alcohol craving was numerically greater in a negative mood than a neutral induction condition, providing weak evidence that depression is associated with greater sensitivity to mood-induced alcohol-seeking (Cooney et al. 1997; Owens et al. 2014). More compellingly, we recently demonstrated that negative mood-induced tobacco-seeking was greater in smokers with current major depressive disorder than those without (Hogarth et al. 2017), corroborating an earlier smoking study reporting a similar effect across subclinical depression symptoms (Fucito and Juliano 2009). In the current study, the failure to find that mood-induced alcohol-seeking was associated with AUDIT and BDI scores was presumably due to the student sample containing too few individual at the more severe end of these spectrums.

To conclude, this study found that individuals who reported drinking to cope with negative affect were more sensitive to the motivational impact of depressive statements on goal-directed alcohol-seeking behaviour in an outcome-revaluation procedure. This effect can be explained by incentive learning, where the negative mood state raises the expected value of alcohol promoting goal-directed alcohol-seeking, but not by S-R habit theory. We have drawn upon wider literature to argue that the development of alcohol dependence, vulnerability to relapse, and the persistence of alcohol use despite substantial costs and intention to quit may be better explained by excessive affective incentive learning than by propensity to habitual or automatic control over alcohol-seeking behaviour.
